# Predictive Value of Preoperative Endometrial Thickness for Concurrent Endometrial Carcinoma in Patients with Endometrial Intraepithelial Neoplasia

**DOI:** 10.3390/jcm15155770

**Published:** 2026-07-23

**Authors:** Şahin Yüksek, Mehmet Sait Bakir, Hasan Turan, Osman Dogan, Numan Bilgic, Murside Cevikoglu Killi

**Affiliations:** Department of Gynecologic Oncology, Mersin City Training and Research Hospital, Mersin 33240, Türkiye; sabakcil@gmail.com (M.S.B.); hasanturan@gmail.com (H.T.); dogano61deniz@gmail.com (O.D.); drnumanbilgic@gmail.com (N.B.); mursidecevikoglu@hotmail.com (M.C.K.)

**Keywords:** endometrial intraepithelial neoplasia, endometrial thickness, endometrial cancer, transvaginal ultrasonography

## Abstract

**Objective.** Endometrial intraepithelial neoplasia (EIN) is a premalignant lesion associated with a substantial risk of concurrent endometrial carcinoma. This study aimed to evaluate whether preoperative endometrial thickness measured by transvaginal ultrasonography could predict concurrent endometrial carcinoma in patients diagnosed with EIN. **Methods.** This retrospective study included 102 patients diagnosed with EIN on endometrial sampling who subsequently underwent hysterectomy. Demographic and clinical data and preoperative endometrial thickness were collected, and final histopathology after hysterectomy was recorded. Receiver operating characteristic (ROC) curve analysis and multivariable logistic regression were performed. **Results.** The median endometrial thickness was 13 mm (range 4–45), and most patients were postmenopausal (68.6%). Final pathology revealed endometrial carcinoma in 50 patients (49.0%) and persistent EIN in 52 (51.0%). Endometrial thickness significantly predicted concurrent carcinoma (AUC 0.679, 95% CI 0.575–0.784; *p* = 0.002), with an optimal cut-off of 14.5 mm (sensitivity 58.0%, specificity 75.0%). Carcinoma was found in 69.0% of patients with thickness ≥ 14.5 mm versus 35.0% below this value (*p* < 0.001). On multivariable analysis, endometrial thickness (OR 1.15 per mm; 95% CI 1.05–1.24; *p* = 0.001) and age (OR 1.11; 95% CI 1.02–1.19; *p* = 0.007) were independently associated with concurrent carcinoma. **Conclusions.** Preoperative endometrial thickness is independently associated with concurrent endometrial carcinoma in patients diagnosed with EIN. A thickness of 14.5 mm or greater may indicate a higher risk of malignancy; however, given its moderate diagnostic accuracy, it should complement rather than replace a comprehensive preoperative assessment.

## 1. Introduction

Endometrial cancer is the most common gynecologic malignancy in developed countries, and its incidence has increased steadily over the past decades. Globally, an estimated 420,000 new cases of endometrial (uterine corpus) cancer were diagnosed in 2022, and its incidence continues to rise, in part reflecting the increasing prevalence of obesity [1]. Abnormal uterine bleeding represents the most frequent presenting symptom and occurs in more than 90% of postmenopausal women with endometrial carcinoma. Transvaginal ultrasonography (TVUS) plays a crucial role in the initial evaluation of endometrial pathology, particularly in patients presenting with abnormal uterine bleeding [2].

Endometrial intraepithelial neoplasia (EIN), previously referred to as complex atypical hyperplasia, is a premalignant lesion of the endometrium associated with a substantial risk of progression to endometrial carcinoma. Previous studies have reported that concurrent endometrial cancer is detected in approximately 30–40% of patients diagnosed with EIN who subsequently undergo hysterectomy [3,4].

EIN represents a monoclonal proliferation of genetically altered endometrial glands with a markedly increased potential for malignant transformation, and in the current World Health Organization classification the terms EIN and atypical endometrial hyperplasia are used synonymously. Its development is strongly linked to prolonged unopposed estrogenic stimulation, and the principal risk factors—obesity, nulliparity, chronic anovulation, diabetes mellitus, and exogenous estrogen exposure—largely overlap with those of endometrioid endometrial carcinoma, underscoring the biological continuum between the two entities. Because endometrial sampling by biopsy or curettage evaluates only a limited portion of the endometrial cavity, a focal coexisting carcinoma may be missed preoperatively; this sampling limitation partly explains the considerable rate of occult malignancy detected only after hysterectomy and provides the rationale for seeking reliable preoperative predictors of concurrent disease.

Given this considerable risk of coexisting malignancy, hysterectomy is considered the standard treatment for patients diagnosed with EIN. However, predicting the presence of concurrent endometrial cancer before surgery remains challenging. Identifying preoperative predictors could improve patient triage and assist in determining whether surgical management should be performed by a gynecologic oncologist and whether additional staging procedures such as lymph node assessment may be required [5,6].

Transvaginal ultrasonography is widely used as a noninvasive diagnostic tool to evaluate the endometrium. Among ultrasound parameters, endometrial thickness has been extensively investigated as a potential predictor of endometrial pathology. Studies have shown that a thicker endometrial stripe is associated with an increased risk of endometrial cancer, although the optimal threshold remains uncertain [7,8].

Several studies have investigated preoperative predictors of concurrent endometrial carcinoma in patients with EIN; however, no clear consensus has yet emerged regarding the predictive value and optimal cut-off of endometrial thickness for detecting concurrent cancer in these patients.

The aim of this study was to evaluate whether preoperative endometrial thickness measured by transvaginal ultrasonography could predict concurrent endometrial carcinoma in patients diagnosed with endometrial intraepithelial neoplasia.

## 2. Materials and Methods

### 2.1. Study Design and Setting

This retrospective cohort study was conducted at the Department of Gynecologic Oncology, Mersin City Training and Research Hospital, Mersin, Türkiye. The study included 102 patients diagnosed with EIN. Medical records of patients operated on between January 2023 and December 2025 were reviewed. The study was approved by the local Ethics Committee of Mersin City Training and Research Hospital (approval number: 2026/167; date of approval: 25 March 2026). and was conducted in accordance with the principles of the Declaration of Helsinki.

### 2.2. Study Population

Patients were eligible for inclusion if they had a histopathological diagnosis of endometrial intraepithelial neoplasia (EIN) on preoperative endometrial biopsy/curettage and subsequently underwent hysterectomy with available final histopathological evaluation. Patients were excluded if they had a preoperative biopsy result other than EIN, no hysterectomy performed, or incomplete clinical, ultrasonographic, or pathological data.

### 2.3. Data Collection

Demographic, clinical, ultrasonographic, and pathological data were extracted from the hospital records. The following variables were recorded: age, body mass index (BMI), parity, menopausal status, presenting symptoms, method of endometrial sampling, preoperative endometrial thickness, preoperative pathology result, surgical approach, and postoperative final pathology result. BMI was calculated as body weight in kilograms divided by the square of height in meters (kg/m^2^). Menopausal status was categorized as premenopausal or postmenopausal, with postmenopausal status defined as at least 12 consecutive months of amenorrhea, and the presenting symptom was classified as postmenopausal bleeding, abnormal uterine bleeding, or other. Endometrial sampling was performed by dilation and curettage or by hysteroscopy-guided sampling, and all preoperative and hysterectomy specimens were evaluated by pathologists according to the World Health Organization criteria. The primary outcome variable of the study was the presence of concurrent endometrial carcinoma in the hysterectomy specimen.

### 2.4. Ultrasonographic Assessment

Preoperative endometrial thickness was assessed by transvaginal ultrasonography (TVUS). Sonographic evaluation was performed by five gynecologic oncologists experienced in transvaginal ultrasonography (HT, MSB, OD, SY, MCK). Endometrial thickness was measured according to standard sonographic principles in the mid-sagittal long-axis view of the uterus. The calipers were placed at the outer margins of the echogenic endometrial borders, and the measurement was taken at the thickest visible portion of the endometrium as the maximum anteroposterior diameter, perpendicular to the endometrial longitudinal axis. Both endometrial layers were included in the measurement. In cases with intracavitary fluid, each single endometrial layer was measured separately and the sum of both layers was recorded. When the endometrium could not be clearly delineated, the measurement was considered not assessable. This measurement approach is consistent with the IETA examination technique and ACOG recommendations for endometrial thickness assessment [2,7,8]. All sonographic examinations were performed transvaginally with a high-frequency endovaginal probe during the preoperative work-up, before hysterectomy; the sonographers were inherently unaware of the final hysterectomy pathology at the time of measurement. Sonographic features that may suggest endometrial malignancy according to the International Endometrial Tumor Analysis (IETA) terminology include a thickened, non-uniform or heterogeneous endometrial echogenicity, an irregular endometrial–myometrial junction, and increased or irregular intralesional vascularity on color Doppler (a high color score) [7,8]. Because the present analysis was based on retrospective records, endometrial thickness was the only sonographic parameter systematically available for all patients and was therefore used as the primary variable of interest; the other IETA morphological and Doppler features were not consistently documented and could not be analyzed.

### 2.5. Histopathological Evaluation

All patients had a preoperative diagnosis of EIN (corresponding to atypical endometrial hyperplasia in the World Health Organization classification) based on endometrial sampling. Final histopathological diagnosis was established from hysterectomy specimens and categorized as either persistent EIN or endometrial carcinoma. The presence of endometrial carcinoma in the hysterectomy specimen was accepted as concurrent malignancy. By study design, all cases with a final diagnosis of carcinoma therefore arose in the setting of a preoperative atypical hyperplasia/EIN diagnosis.

### 2.6. Statistical Analysis

Statistical analysis was performed using IBM SPSS Statistics for Windows (version 23; IBM Corp., Armonk, NY, USA). Continuous variables were assessed for distributional characteristics using the Kolmogorov–Smirnov test; normally distributed variables were expressed as mean ± standard deviation (SD) and non-normally distributed variables as median (range). Categorical variables were presented as number (n) and percentage (%), categorical variables using the chi-square or Fisher exact test. To evaluate the diagnostic performance of preoperative endometrial thickness for predicting concurrent endometrial carcinoma, receiver operating characteristic (ROC) curve analysis was performed and the area under the curve (AUC) was calculated. The statistical significance of the ROC curve was tested against the null hypothesis of AUC = 0.50. The optimal cut-off value was determined using the Youden index, and the corresponding sensitivity and specificity were reported, together with the positive and negative predictive values and likelihood ratios; 95% confidence intervals for sensitivity and specificity were calculated using the Wilson method. Patients were stratified according to the optimal cut-off value, and the distribution of final pathology categories between groups was compared using the chi-square test. Variables associated with concurrent carcinoma were further evaluated using multivariable binary logistic regression (including age, BMI, menopausal status, and endometrial thickness), and odds ratios (OR) with 95% confidence intervals (CI) were reported. A two-sided *p* value < 0.05 was considered statistically significant.

## 3. Results

A total of 102 patients diagnosed with endometrial intraepithelial neoplasia (EIN) who underwent hysterectomy were included in the study.

### 3.1. Patient Characteristics

The demographic and clinical characteristics of the study population are summarized in Table 1. The mean age of the patients was 56.7 ± 10.9 years, and the mean body mass index was 35.1 ± 6.4 kg/m^2^. The majority of patients were postmenopausal (68.6%), while 31.4% were premenopausal. Postmenopausal bleeding was the most common presenting symptom (66.7%), followed by abnormal uterine bleeding (26.4%). Endometrial sampling was performed by dilation and curettage in 66.7% and by hysteroscopy in 33.3% of patients. The median preoperative endometrial thickness measured by transvaginal ultrasonography was 13 mm (range 4–45). Hysterectomy was performed by a laparoscopic approach in 89.2% of cases.

### 3.2. Final Histopathological Outcomes

Final hysterectomy pathology revealed endometrial carcinoma in 50 patients (49.0%) and persistent EIN in 52 patients (51.0%).

### 3.3. Association Between Endometrial Thickness and Concurrent Carcinoma

Patients were stratified according to the optimal cut-off value of endometrial thickness determined by ROC analysis (14.5 mm). Among patients with an endometrial thickness < 14.5 mm, endometrial carcinoma was detected in 21 of 60 patients (35.0%). In contrast, among patients with an endometrial thickness ≥ 14.5 mm, endometrial carcinoma was identified in 29 of 42 patients (69.0%). This difference was statistically significant (*p* < 0.001) (Table 2).

### 3.4. ROC Analysis

**Figure 1:**
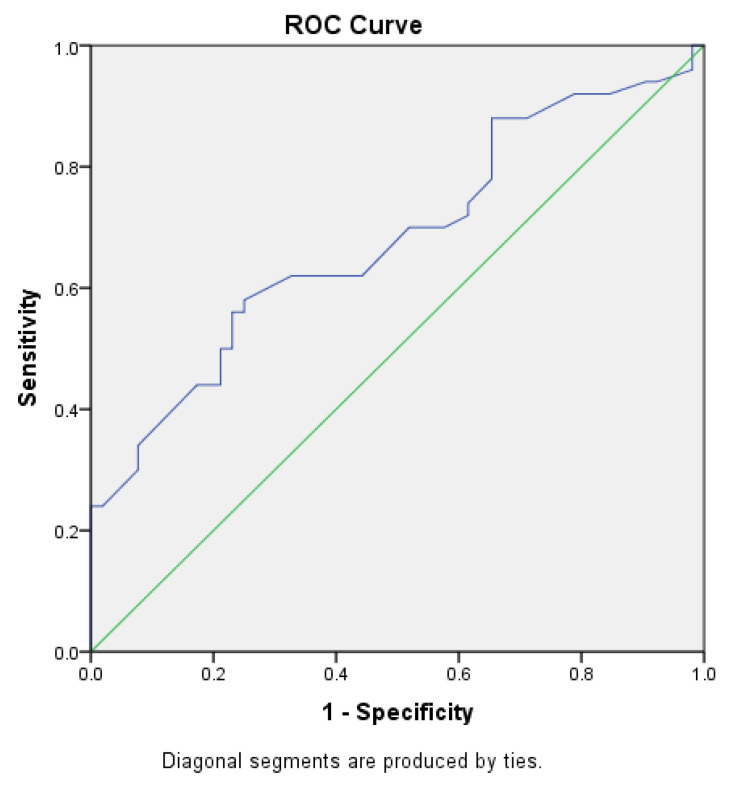
ROC curve of endometrial thickness predicting concurrent endometrial carcinoma.

Receiver operating characteristic (ROC) curve analysis demonstrated that preoperative endometrial thickness significantly predicted concurrent endometrial carcinoma (AUC = 0.679, 95% CI 0.575–0.784, *p* = 0.002). The optimal cut-off value determined by the Youden index was 14.5 mm, yielding a sensitivity of 58.0% and a specificity of 75.0% for predicting concurrent endometrial carcinoma (Table 3, Figure 1).

At the 14.5 mm threshold, the sensitivity was 58.0% (95% CI 44.2–70.6) and the specificity was 75.0% (95% CI 61.8–84.8). The positive predictive value was 69.0% (95% CI 54.0–80.9) and the negative predictive value was 65.0% (95% CI 52.4–75.8), with a positive likelihood ratio of 2.32, a negative likelihood ratio of 0.56, and a Youden index of 0.33. Because the specificity was moderate, approximately one-quarter of patients without carcinoma (13 of 52, 25.0%) were classified above the threshold, corresponding to a false-positive rate of 25.0% (Table 3).

### 3.5. Multivariable Analysis

In the multivariable logistic regression model, endometrial thickness remained an independent predictor of concurrent endometrial carcinoma (OR 1.15 per mm; 95% CI 1.05–1.24; *p* = 0.001), as did age (OR 1.11 per year; 95% CI 1.02–1.19; *p* = 0.007). Menopausal status (OR 1.37; 95% CI 0.29–6.45; *p* = 0.683) and BMI (OR 0.99; 95% CI 0.93–1.06; *p* = 0.934) were not significantly associated with concurrent carcinoma (Table 4). These findings indicate that increased preoperative endometrial thickness is independently associated with a significantly higher risk of concurrent endometrial carcinoma in patients diagnosed with EIN.

## 4. Discussion

In this study, we evaluated the predictive value of preoperative endometrial thickness measured by transvaginal ultrasonography for detecting concurrent endometrial carcinoma in patients diagnosed with endometrial intraepithelial neoplasia. Our findings demonstrated that increased endometrial thickness was significantly and independently associated with the presence of concurrent endometrial carcinoma. ROC analysis revealed a moderate predictive performance (AUC = 0.679), and an endometrial thickness cut-off value of 14.5 mm provided the optimal balance between sensitivity and specificity.

Endometrial intraepithelial neoplasia is widely recognized as a premalignant lesion with a substantial risk of concurrent endometrial carcinoma at the time of hysterectomy. Previous studies have reported concurrent carcinoma rates ranging from approximately 30% to 40%, emphasizing the importance of definitive surgical management [3]. In the present study, concurrent endometrial carcinoma was identified in 49.0% of patients. Although slightly higher than many published series, this finding is consistent with reports suggesting that a considerable proportion of patients diagnosed with EIN may harbor occult carcinoma at the time of hysterectomy.

Identifying reliable preoperative predictors of concurrent malignancy remains clinically important. Accurate risk stratification may guide referral patterns and surgical planning, particularly regarding the involvement of gynecologic oncologists and the potential need for lymph node assessment. Vetter et al. demonstrated that patients with concurrent endometrial carcinoma had significantly greater endometrial thickness than those without malignancy, indicating that ultrasound evaluation may provide valuable risk stratification [3,4]. Similarly, Abt et al. evaluated a large cohort of patients with EIN and reported that an endometrial stripe ≥ 15 mm was associated with a significantly increased risk of malignancy and was also linked to high-risk pathological features [5].

Our results are consistent with these findings. In the present study, patients with an endometrial thickness ≥ 14.5 mm had a markedly higher prevalence of concurrent carcinoma compared with those with thinner endometrium (69.0% vs. 35.0%). Moreover, endometrial thickness remained an independent predictor of concurrent carcinoma in multivariable analysis (OR 1.15 per mm; *p* = 0.001), supporting the concept that increasing endometrial thickness may reflect underlying malignant transformation or more advanced premalignant disease. A comparison of endometrial thickness cut-off values reported in the literature is presented in Table 4; our optimal threshold of 14.5 mm falls within the range described in previous EIN cohorts (≈11–20 mm).

Beyond the studies of Vetter et al. and Abt et al., other reports summarized in Table 5 have proposed a range of thresholds. Burrows et al., in 126 women with atypical hyperplasia, found that an endometrial thickness greater than 11 mm was associated with approximately six-fold higher odds of concurrent carcinoma (OR 6.1) [9], whereas recent series from Türkiye reported lower thresholds: Aytekin et al., in 196 patients with EIN, identified a significant association at ≥13 mm [10], and Çaltek et al. described an approximately four-fold increased risk at ≥14 mm [11]. The variation in optimal cut-offs across these studies (≈11–20 mm) likely reflects differences in population characteristics, measurement technique, and the prevalence of concurrent carcinoma, and reinforces that a single universal threshold is unlikely to be applicable to all settings. We found that when the threshold value of 14.5 was taken as the cut-off in our study, it predicted approximately 4 times the incidence of endometrial cancer, which is consistent with previous studies in the literature (OR: 4.14 (95% CI; 1.78–9.61, *p* = 0.001, Table 5). Our threshold of 14.5 mm is broadly consistent with these contemporary cohorts, particularly the recent Turkish series.

Transvaginal ultrasonography remains a fundamental tool for evaluating endometrial pathology. Several studies have demonstrated that endometrial thickness measurements are associated with the risk of endometrial malignancy, particularly in women presenting with abnormal uterine bleeding [7,8]. However, the diagnostic performance of endometrial thickness varies depending on patient population and clinical context. In patients with a known diagnosis of EIN, determining the optimal cut-off value for predicting concurrent cancer remains challenging. Our ROC analysis identified 14.5 mm as the optimal threshold, with 58.0% sensitivity and 75.0% specificity, suggesting moderate diagnostic accuracy.

These diagnostic performance measures should be interpreted with caution. Although endometrial thickness was significantly and independently associated with concurrent carcinoma, the AUC of 0.679 reflects only moderate discriminatory ability, indicating substantial overlap in thickness measurements between malignant and non-malignant cases. The moderate positive and negative predictive values (69.0% and 65.0%, respectively) and the modest likelihood ratios (positive likelihood ratio 2.32; negative likelihood ratio 0.56) indicate that endometrial thickness alone cannot reliably confirm or exclude concurrent carcinoma. Endometrial thickness is therefore best regarded as one component of a broader clinical assessment rather than a stand-alone predictive tool for surgical decision-making.

It should also be acknowledged that endometrial thickness is a nonspecific sonographic marker that may reflect benign hyperplasia, hormonal influences, the burden of intraepithelial neoplasia, or malignancy. Accordingly, the observed association between increased thickness and concurrent carcinoma does not establish a direct mechanistic link, and the moderate AUC is consistent with considerable overlap in measurements across these underlying conditions.

In particular, because the sensitivity at the 14.5 mm threshold was only 58.0%, a value below this cut-off cannot reliably exclude concurrent carcinoma, and a substantial proportion of malignancies occurred in patients with thinner endometrium. Rather than a definitive predictor, endometrial thickness is more appropriately viewed as a clinically useful parameter that helps differentiate patients at higher versus lower risk of concurrent malignancy, the performance of which is strongly dependent on population characteristics such as age, body mass index, menopausal status, and the presence of abnormal uterine bleeding.

Despite these findings, the predictive value of clinical and imaging parameters for concurrent endometrial carcinoma remains limited. A recent study using machine learning models attempted to improve prediction accuracy by integrating multiple clinical and pathological variables, including endometrial thickness. However, even advanced algorithms demonstrated only modest predictive performance, highlighting the difficulty of reliably identifying concurrent carcinoma preoperatively [6]. These findings underscore the importance of maintaining a high index of suspicion and continuing to consider hysterectomy as the definitive management for patients with EIN.

The clinical implications of our findings are relevant for surgical planning. Since a significant proportion of patients with EIN may harbor occult carcinoma, identifying those at higher risk could help determine whether surgery should be performed by a gynecologic oncologist and whether intraoperative staging procedures might be necessary. Our findings suggest that patients with endometrial thickness ≥ 14.5 mm may represent a subgroup with increased risk of concurrent carcinoma and may benefit from more careful preoperative assessment. The rate of concurrent endometrial carcinoma observed in our study (49.0%) is comparable to rates reported in previous studies; for example, Vetter et al. reported a concurrent carcinoma rate of approximately 48.5%, while Abt et al. identified invasive carcinoma in 27% of patients and Levin et al. reported a prevalence of approximately 37.5% [3,5,6].

Because dichotomizing a continuous measurement at a single threshold discards prognostic information, we also analyzed endometrial thickness as a continuous variable; each 1 mm increase was independently associated with a 15% increase in the odds of concurrent carcinoma (OR 1.15; 95% CI 1.05–1.24). Nevertheless, applying a fixed 14.5 mm cut-off misclassified approximately one quarter of patients without carcinoma as test-positive (false-positive rate 25.0%). In clinical terms, this could translate into more extensive surgery or referral for a proportion of women who do not harbor malignancy, and this potential for overtreatment must be weighed against the benefit of identifying occult carcinoma.

These considerations should be interpreted in the context of current guideline recommendations. The ACOG Clinical Consensus recommend Endometrial intraepithelial neoplasia (EIN) or atypical endometrial hyperplasia (AEH) often is a precursor lesion to adenocarcinoma of the endometrium. Hysterectomy is the definitive treatment for EIN-AEH. When a conservative (fertility-sparing) approach to the management of EIN-AEH is under consideration, it is important to attempt to exclude the presence of endometrial cancer to avoid potential undertreatment of an unknown malignancy in those who have been already diagnosed with EIN-AEH. Given the high risk of progression to cancer, those who do not have surgery require progestin therapy (oral, intrauterine, or combined) and close surveillance [12]. Similarly, the ESGO/ESTRO/ESP guidelines emphasize individualized preoperative risk assessment and appropriate referral to gynecologic oncologists when occult carcinoma is suspected, while the FIGO recommendations reinforce comprehensive surgical planning according to estimated oncologic risk rather than relying on a single preoperative parameter [13,14]. Since preoperative sampling cannot reliably exclude concurrent carcinoma, a low threshold for gynecologic-oncology involvement is prudent; our observation that a substantial proportion of malignancies occurred even below the 14.5 mm threshold reinforces that endometrial thickness should complement, rather than replace, this guideline-based approach to surgical planning and staging.

Our concurrent-cancer rate of 49.0% lies at the higher end of the published spectrum. A systematic review and meta-analysis of women with atypical hyperplasia reported a pooled concurrent endometrial cancer prevalence of approximately 32.6% (95% CI 24.1–42.4%) [15], whereas single-institution cohorts applying strict endometrial intraepithelial neoplasia criteria have reported rates approaching 47% when the lesion is confined to the endometrium rather than to a polyp [16]. The relatively high rate in our cohort—composed exclusively of patients proceeding to hysterectomy—is consistent with these endometrium-confined series and reinforces that occult carcinoma is common in this population. This observation further supports current international recommendations advocating meticulous preoperative evaluation before definitive surgery [13,14,17].

These observations also inform the ongoing debate regarding nodal assessment. A recent meta-analysis of surgical nodal evaluation in atypical hyperplasia/EIN found that, although approximately 44% of patients were upstaged to carcinoma after hysterectomy, the rate of lymph-node metastasis was below 2% [18]. Routine sentinel lymph node biopsy would therefore overtreat most patients, and current evidence favors a selective, risk-adapted approach in which preoperative markers—including endometrial thickness together with age, body mass index and menopausal status—help identify the minority who may benefit from concurrent staging [19]. Current NCCN recommendations and recent systematic reviews support a selective, risk-adapted approach in which sentinel lymph-node evaluation is considered only for patients with clinical, radiological or intraoperative findings suggestive of invasive disease [17,20]. Our finding that an endometrial thickness ≥ 14.5 mm identifies a subgroup with a 69% prevalence of concurrent carcinoma supports incorporating this simple sonographic parameter into such risk-adapted algorithms, while recognizing that it cannot, by itself, determine the need for lymph node assessment. Nevertheless, because of its only moderate discriminatory performance, it should always be interpreted together with clinical characteristics, pathological findings and guideline-based surgical decision making.

From a clinical-application standpoint, an inexpensive and widely available parameter such as endometrial thickness could be embedded in preoperative triage pathways to help decide which patients with EIN are referred to a gynecologic oncologist and counselled about the possible need for concurrent surgical staging. Future research should focus on prospective, multicenter studies to externally validate the 14.5 mm threshold across populations with differing body mass index distributions, and on the development of multimodal prediction models that integrate endometrial thickness with color Doppler and other IETA morphological features, clinical risk factors (age, body mass index, menopausal status, diabetes), and emerging molecular, biomarker, or radiomic data. Machine-learning approaches applied to such combined datasets may further refine individualized risk estimation and, ultimately, reduce both the underdetection of occult carcinoma and the overtreatment of patients with truly benign disease.

This study has several limitations. First, the retrospective design may introduce selection bias and limit the availability of certain clinical variables. As a single-center study, it may also be subject to institutional selection bias, and the findings should ideally be validated in independent, multicenter cohorts. Second, the relatively small sample size may restrict the generalizability of the findings. Third, ultrasonographic measurements were performed by multiple operators, which may introduce interobserver variability, although all examinations were performed by experienced gynecologic oncologists. Because of the retrospective design, formal interobserver agreement (for example, the intraclass correlation coefficient) among the five sonographers could not be quantified, which represents an additional limitation. Fourth, endometrial thickness was the only sonographic parameter analyzed; other features suggestive of malignancy, such as endometrial echogenicity, the endometrial–myometrial junction, and color Doppler vascularity, were not systematically recorded and could not be evaluated. Prospective studies incorporating these IETA morphological and vascular parameters, potentially in a multimodal predictive model, may further improve preoperative risk stratification. Fifth, the study population was predominantly obese (mean body mass index 35.1 kg/m^2^); as elevated body mass index may influence both the sonographic measurement of endometrial thickness and the underlying risk of endometrial cancer, our findings and the proposed threshold may not be fully generalizable to non-obese populations. Despite these limitations, the study also has several strengths. The use of hysterectomy specimens as the reference standard allowed accurate identification of concurrent malignancy, and all patients had a confirmed diagnosis of EIN prior to surgery, allowing a focused evaluation of this clinically important population.

## 5. Conclusions

Preoperative endometrial thickness measured by transvaginal ultrasonography is independently associated with, and clinically useful for identifying, concurrent endometrial carcinoma in patients diagnosed with endometrial intraepithelial neoplasia. In our cohort, an endometrial thickness of 14.5 mm or greater was associated with a substantially increased risk of concurrent malignancy. These findings suggest that increased endometrial thickness may help identify patients at higher risk for underlying carcinoma and may assist in preoperative risk stratification and surgical planning. However, given its moderate diagnostic accuracy and limited specificity, endometrial thickness should be interpreted as one component of a comprehensive preoperative clinical assessment rather than a stand-alone criterion for surgical decision-making. Further prospective studies with larger sample sizes are warranted to validate these findings and to better define the role of ultrasonographic parameters in predicting concurrent malignancy in patients with EIN.

## Figures and Tables

**Table 1:** Demographic and clinical characteristics of the study population (*n* = 102).

Characteristic	Category	Value
Age (years)	mean ± SD	56.7 ± 10.9
BMI (kg/m^2^)	mean ± SD	35.1 ± 6.4
Endometrial thickness (mm)	median (range)	13 (4–45)
Parity	median (range)	2 (0–8)
Menopausal status	Premenopausal	32 (31.4%)
	Postmenopausal	70 (68.6%)
Presenting symptom	Postmenopausal bleeding	68 (66.7%)
	Abnormal uterine bleeding	27 (26.4%)
	Other	7 (6.9%)
Diagnostic method	Dilation and curettage	68 (66.7%)
	Hysteroscopy	34 (33.3%)
Surgical approach	Laparoscopy	91 (89.2%)
	Laparotomy	11 (10.8%)

**Table 2:** Association between endometrial thickness and final histopathology.

Endometrial Thickness	Persistent EIN, *n* (%)	Endometrial Carcinoma, *n* (%)	*p* Value
<14.5 mm (*n* = 60)	39 (65.0)	21 (35.0)	<0.001
≥14.5 mm (*n* = 42)	13 (31.0)	29 (69.0)	<0.001
**Total (*n* = 102)**	**52 (51.0)**	**50 (49.0)**	**<0.001**

Percentages are calculated within each thickness group (row percentages). *p* value by chi-square test. EIN, endometrial intraepithelial neoplasia.

**Table 3:** ROC analysis results for endometrial thickness predicting concurrent endometrial carcinoma.

AUC (95% CI)	*p* Value	Cut-Off (mm)	Sensitivity (%)	Specificity (%)
0.679 (0.575–0.784)	0.002	14.5	58.0	75.0

At the 14.5 mm cut-off: sensitivity 58.0% (95% CI 44.2–70.6), specificity 75.0% (95% CI 61.8–84.8), positive predictive value 69.0% (95% CI 54.0–80.9), negative predictive value 65.0% (95% CI 52.4–75.8), positive likelihood ratio 2.32, negative likelihood ratio 0.56, Youden index 0.33. AUC, area under the curve; CI, confidence interval. Optimal cut-off determined by the Youden index.

**Table 4:** Multivariable logistic regression analysis of factors associated with concurrent endometrial carcinoma.

Variable	OR	95% CI	*p* Value
Age (per year)	1.11	1.02–1.19	0.007
Endometrial thickness (per mm)	1.15	1.05–1.24	0.001
Menopausal status (postmenopausal)	1.37	0.29–6.45	0.683
BMI (per kg/m^2^)	0.99	0.93–1.06	0.934

OR, odds ratio; CI, confidence interval; BMI, body mass index.

**Table 5:** Studies evaluating endometrial thickness cut-off values for predicting concurrent endometrial carcinoma in EIN.

Study	Year	Sample Size	Population	Cut-Off (mm)	Main Finding
Vetter et al. [3]	2020	169	EIN undergoing hysterectomy	≥20	≈4-fold increased risk of concurrent EC
Abt et al. [5]	2022	378	EIN	≥15	Higher risk of EC and high-risk pathology
Burrows et al. [9]	2021	126	AEH	>11	≈6-fold increased odds of EC (OR 6.1)
Aytekin et al. [10]	2025	196	EIN	≥13	Significant association with concurrent EC
Çaltek et al. [11]	2025	172	EIN	≥14	≈4-fold increased EC risk (aOR 4.06)
Present study	2026	102	EIN	≥14.5	≈4-fold increased odds of EC (OR 4.1) Sensitivity 58%, specificity 75%

EC, endometrial carcinoma; EIN, endometrial intraepithelial neoplasia.

## Data Availability

The data presented in this study are available on request from the corresponding author.

## Abbreviations

EIN: endometrial intraepithelial neoplasia; EC, endometrial carcinoma; TVUS, transvaginal ultrasonography; ROC, receiver operating characteristic; AUC, area under the curve; BMI, body mass index; IETA, International Endometrial Tumor Analysis; SLN, sentinel lymph node; OR, odds ratio; CI, confidence interval.
